# Non-adherence to inhaled medications among adult asthmatic patients in Ethiopia: a systematic review and meta-analysis

**DOI:** 10.1186/s40733-020-00065-7

**Published:** 2020-10-14

**Authors:** Woldu Aberhe, Abrha Hailay, Kidane Zereabruk, Guesh Mebrahtom, Teklehaimanot Haile

**Affiliations:** 1 Department of Adult Health Nursing, School of Nursing, Aksum University, Aksum, Ethiopia; 2 Department of Maternity and Neonatal Nursing, School of Nursing, Aksum University, Aksum, Ethiopia

**Keywords:** Asthma, Ethiopia, Medications non-adherence, Meta-analysis, And prevalence

## Abstract

**Background:**

Medication non-adherence is one of a common problem in asthma management and it is the main factor for uncontrolled asthma. It can result in poor asthma control, which leads to decreased quality of life, increase hospital admission, increased health care utilization, lost productivity, and mortality. To date, there have been no studies and protocols that estimated the pooled national prevalence of non-adherence to inhaled anti-asthmatic medications in Ethiopia. Therefore, the primary purpose of this systematic review and meta-analysis is to determine the pooled national prevalence of non-adherence to inhaled medications among asthmatic patients in Ethiopia.

**Methods:**

Different database searching engines including PubMed, Scopus, Google Scholar, Africa journal online, World Health Organization afro library, and Cochrane review were systematically searched by using keywords such as “prevalence, non-adherence to inhaled medications, inhaled corticosteroids, and asthmatic patients” and their combinations. Six published observational studies that report the prevalence of non-adherence to inhaled medications were finally selected. The Preferred Reporting Items for Systematic Review and Meta-Analysis guideline was followed. Heterogeneity across the included studies was evaluated by the inconsistency index (I^2^). The random-effect model was fitted to estimate the pooled prevalence of non-adherence to inhale anti-asthmatic medications. All statistical analysis was done using R version 3.5.3 and R Studio version 1.2.5033 software for windows.

**Results:**

The pooled national prevalence of non-adherence to inhaled medications among asthmatic patients was 29.95% (95% CI, 19.1, 40.8%). The result of this meta-analysis using the random-effects model revealed that there is high heterogeneity across the included studies. The result of subgroup analysis indicates that one out of three in the Oromia region and one out of five in the Amhara region asthmatic patients was non-adherent to their inhaled anti-asthmatic medications.

**Conclusion:**

the prevalence of non-adherence to inhaled anti-asthmatic medications was high. Thus, our finding suggests that one out of four asthmatic patients were non-adherent to inhaled medications. The ministry of health, health policymakers, clinicians, and other health care providers should pay attention to strengthening the adherence levels to inhaled anti-asthmatic medications, and country-based interventions should be developed to reduce the burden of non-adherence to inhaled anti-asthmatic medications.

## Background

Asthma is a major chronic respiratory disease that affects around 339 million people worldwide [1]. There has been an increase in the global prevalence, economic burden, morbidity, and mortality associated with asthma over the last four decades. Globally, asthma ranked 28th among the leading causes of the burden of disease and 27th in low- and middle-income countries [1, 2]. Despite Ethiopia was signed to achieve a sustainable development goal to reduce the premature death from non-communicable diseases by one third from 2016 to 2030 but the annual death of the Ethiopia population due to non-communicable diseases such as asthma is still high (39%) [3, 4]. In Ethiopia, the world health organization reported that 1.12% of the total deaths were caused by asthma and ranked 18th from the world [5].

Adherence is ‘the extent to which a patient’s behavior corresponds with recommendations from a health care provider’ [6]. Inhaled medicines such as inhaled corticosteroids are the therapeutic use of inhaled gases that the patients breathe directly (through inhalers) into the lungs for treatment of chronic lung disease [7]. Despite the introduction of inhaled medications as the primary treatment for asthma has led to significant improvements in asthma control [1, 8], Medication non-adherence is still one of a common problem in asthma management. Adherence to asthma medication is very poor, with the prevalence of non-adherence ranging from 30 to 70%. The annual economic burden of asthma in developing countries was over $20 billion and up to three-quarters of the total costs associated with asthma are due to poor asthma control [9–13]. Non-adherence to inhaled corticosteroids is likely responsible for 24% asthma exacerbations [14]. It can result in poor asthma control, which leads to decreased quality of life, increase hospital admission, increased health care utilization, lost productivity, and mortality [15–17].

Although several studies have been published on the prevalence and associated factors of non-adherence to inhaled medications among adult asthmatic patients in Ethiopia [18–23], the general prevalence remains unknown. Therefore, the primary purpose of this systematic review and meta-analysis is to determine the pooled national prevalence of non-adherence to inhaled medications among adult asthmatic patients in Ethiopia.

### Objective

This study aimed to estimate the pooled national prevalence of non-adherence to inhaled medications among adult asthmatic patients in Ethiopia.

## Methods

### Search strategy/ information sources

This systematic review and meta-analysis were conducted following the Preferred Reporting Items for Systematic Reviews and Meta-Analyses (PRISMA) guidelines [24]. We searched articles reporting the prevalence of non-adherence to inhaled medications among adult asthmatic patients from (PubMed/MEDLINE, Embase, Science Direct, Scopus, Google Scholar, Web of Science, Cochran library, Africa Wide Information, World Health Organization (WHO) afro library, and Africa Index Medicus) using Electronic databases from inception to September 1/2020. The following keywords were searched by using BOOLEAN (AND/OR) operators to combine search terms. “medication adherence”, “inhaled corticosteroids”, “non-adherence to inhaled medications”, “treatment adherence,” “associated factors”, “asthmatic patients”, “Ethiopia, “systematic review and meta-analysis”, and combinations of these terms were used. The search from the above databases confirmed that there was no systematic review and /or protocol on the topic of interest. Moreover, the reference lists of eligible articles were also searched to retrieve additional relevant articles.

### Data extraction and quality assessment

From each included study, information on the name of the first author, year of publication,

study area (region), health facility, study design, cases, sample size, medication adherence status, response rate, the prevalence of non-adherence to inhaled medications among adult asthmatic patients in Ethiopia were extracted using a pre-piloted template prepared in a Microsoft Excel spreadsheet. The two reviewers (WA and KZ), screened the titles, abstracts of all citations retrieved, and the full-text search results to identify potentially eligible studies. When necessary, authors were contacted for additional information to confirm eligibility of studies. Where there is missing information, the corresponding author of the study was contacted to request the missing information. Emails were sent to the corresponding authors to request for additional information before excluding the study. Disagreements were resolved by discussion after mutual consensus with a third reviewer who is an experienced researcher (AH) in meta-analysis studies. The search results were uploaded to EndNote software first to remove duplicates. Finally, 6 research papers out of 104 articles were selected.

### Criteria for considering studies for the review

#### Inclusion criteria

Design: All published and unpublished observational studies.

Population: Study participants should be at least 18 years of age.

Publication status: Only peer-reviewed articles.

Settings: Hospital-based studies.

Language: The articles included in this study were those articles published only in the English language. This is because of publication through other languages in Ethiopia is uncommon Publication or report year: We reviewed all publications that report the prevalence of non-adherence to inhaled medications among adult asthmatic patients in Ethiopia.

Method of diagnosis: No restriction on methods of diagnosis.

Intervention(s)/exposure(s): On inhaled anti-asthmatic medications.

Outcome: Prevalence of non-adherence to inhaled medications.

#### Exclusion criteria

Observational studies including case reports and case series were excluded. Studies not performed in humans, qualitative studies, studies that lack relevant data needed to compute the prevalence or frequency of non-adherence to inhaled medications, and studies among children.

### Quality assessment of included studies

The methodological quality of the included studies was evaluated using the Newcastle-Ottawa Scale. The Newcastle-Ottawa Scale was designed to assess the quality of non-randomized studies in meta-analyses. This scale is primarily formulated by a star allocation system, assigning a maximum of 10 stars for the risk of bias in three areas: a selection of study groups (4 or 5 stars), comparability of groups (2 stars), and ascertainment of the outcome of interest or the exposure (3 stars). No validation study provides a cut-off score for rating low-quality studies; a priori, we arbitrarily established that 0–3, 4–6 and 7–10 stars would be considered at high, moderate, and low risk of bias, respectively [25].

### Statistical analysis and presentation of results

Data were analyzed using the R version 3.5.3 and Rstudio version 1.2.5003 software. Forest plots were drawn to visualize the combined prevalence of non-adherence to inhaled medications and the extent of statistical heterogeneity between studies. Heterogeneity across the studies was assessed by Cochrane’s Q test and I^2^ statistic. I^2^ statistic ranges from 0 to 100%. I^2^ statistic (with values of 25, 50, and 75% is representative of a low, medium, and high heterogeneity, respectively) [26]. There was high heterogeneity between the included studies. Therefore, we used a random-effects model to estimate the overall pooled national prevalence of non-adherence to inhaled medications. A subgroup analysis was summarized by geographic regions where the study was conducted.

### Data management

Based on the inclusion and exclusion criteria, a tool has been developed a priori to guide the screening and selection process. The tool was piloted and revised before data extraction begins. The search results were uploaded to EndNote software first to remove duplicates.

### Selection process

Once data are obtained, two reviewers independently screen the titles and abstracts of articles retrieved from the literature search against the inclusion criteria. Full texts for the eligible titles and/or abstracts including those where there is uncertainty were obtained for further assessment on whether to include in the study or not. Where necessary, authors were contacted for additional information to confirm eligibility of studies. Disagreements were resolved through discussion and when needed there was arbitration by a third reviewer. Reasons for excluding articles were recorded.

### Data collection process

Data were extracted using a standardized data extraction form. From the studies included, two assessors will independently extract data using the predefined standardized extraction form. Disagreements were resolved through discussion and when needed there was arbitration by a third reviewer.

Where there is missing information, the corresponding author of the study was contacted to request the missing information. Emails were sent to the corresponding author to request for additional information before excluding the study.

### Data items

Data on general information, authors, publication year, country, and region, data collection year, study characteristics (study design, setting, case, sample size, response rate), were extracted.

### Outcomes and prioritization

The primary outcome is the prevalence of non-adherence to inhaled medications among adult asthmatic patients in Ethiopia.

### Risk of bias in individual studies

To assess the risk of bias and quality of studies included in this review, a tool developed by Hoy et al. for prevalence studies were used [27]. The tool contains 11 items; items 1–4 assess the external validity, 5–10 assess the internal validity, and item 11 is a summary of the overall risk by the reviewer based on the responses of the above 10 items which are scored 1 if yes and 0 if no. Studies was classified as having a low (> 8), moderate [28–30], or high (≤ 5) risk of bias. This exercise was done by two reviewers and disagreements were solved by discussion and where necessary by arbitration involving a third reviewer/author (AH).

For each included study, we will estimate the precision (C) or margin of error, considering the sample size (SS) and the observed prevalence (p) of non-adherence to inhaled medications from the formula:

SS= $\frac{z^2\ast p\ast \left(1-p\right)}{d^2}$ where *Z* was the *z* value fixed at 1.96 across studies (corresponding to 95% confidence interval). The desirable margin of error is 5% (0.05) or lower.

### Data synthesis

A meta-analysis was performed to estimate the pooled prevalence of non-adherence to inhaled medications. Results were presented using forest plots. A subgroup analysis was summarized by geographic regions where the study was conducted. A random-effects meta-analysis was performed [31] to determine the pooled estimate prevalence of non-adherence to inhaled medications in Ethiopia. Heterogeneity was explored using Cochrane’s *Q* and quantified by *I*^*2*^ statistics [26]. Results were reported as proportions with corresponding 95% confidence intervals (CIs). The results of this review were reported based on the Preferred Reporting Items for Systematic Reviews and Meta-Analyses (PRISMA) guidelines [32] (Supplementary file 1-PRISMA checklist).

## Results

### Screening flow

**Fig. 1:**
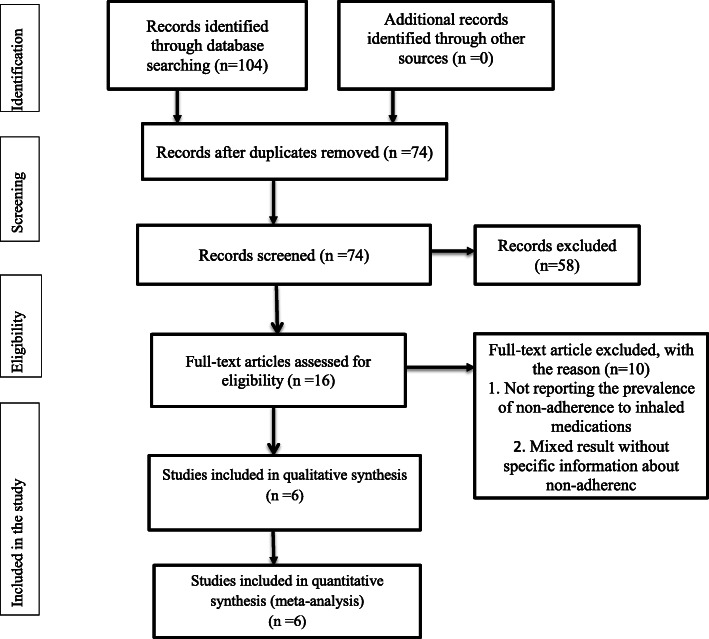
Flow chart diagram for selection of articles for meta-analysis of Non-adherence to inhaled anti-asthmatic medications

Totally 6 articles were reviewed base on the four steps of the PRISMA statement [24]. The online search process initially yielded 104 articles. Of which 30 articles duplicate records were removed. After reviewing the title and abstract, we excluded 58 irrelevant articles. From the remaining 16 articles, 10 articles were excluded since they failed to meet eligibility. Finally, a total of 6 relevant articles with 921 asthmatic patients were included in the meta-analysis. The detailed steps of the screening process are shown in a PRISMA flow chart of the study selection (Fig. 1).

### Study characteristics

In this systematic review and meta-analysis, 6 studies were included. Of the studies, 2(33.3%) were conducted in Oromia, 2(33.3%) in Amara, and the other two were from Addis Ababa and SNNPE. All the included studies were cross-sectional studies with the sample size range from 106 to 242 asthmatic patients. The highest prevalence of non-adherence to inhaled medications was reported from SNNPE (59%) and the lowest prevalence of non-adherence was reported in Addis Ababa (15.1%). The quality score of each primary study, based on the Newcastle Ottawa Scale quality assessment criteria, showed no considerable risk; therefore, all the studies were considered in this systematic review and meta-analysis (Table 1).

**Table 1:** Characteristics of studies included in the systematic review and meta-analysis of the non-adherence to inhaled medications among asthmatic patients in Ethiopia, 2020

Authors	publication year	data collection year	Region	Health Facility Name	SD	cases	Sample size	Quality assessment (Based on NOS)	response rate	Prevalence %
Ayele and Tegegn [16]	2017	2016	Amhara	hospital	CS	30	164	9	100	18.3
Tesfaye et al. [17]	2018	2017	Amhara	hospital	CS	34	150	9	92.60%	22.70%
Zewudie et al. [19]	2019	2018	Oromia	hospital	CC	69	242	8	100%	28.5
Kebede and Mamo [18]	2019	2018	Oromia	hospital	CS	53	140	9	100%	37.9
Koyra and Chinasho [20]	2091	2018	SNNPE	hospital	CS	63	106	7	100%	59
Ayele et al. [21]	2017	2014	A A	hospital	CS	18	119	8	100%	15.1

*AA* Addis Ababa, *SNNPE* South Nation Nationalities and People Ethiopia, *CS* Cross-sectional, *SD* Study Design, *CC* case control

### The pooled prevalence of non-adherence to inhaled medications

**Fig. 2:**
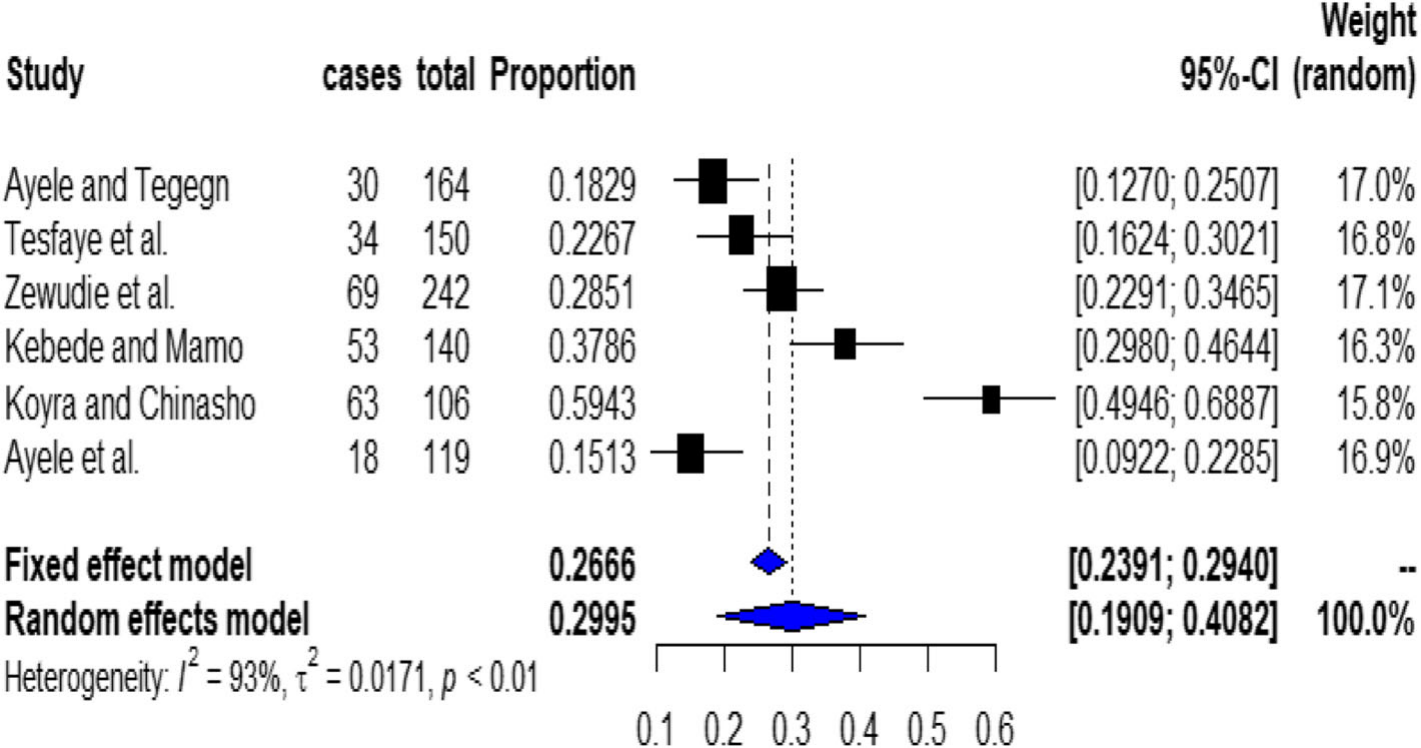
Forest plot for the pooled prevalence of non-adherence to inhaled medications among asthmatic patients from 6 observational studies

The pooled national prevalence of non-adherence to inhaled medications among asthmatic patients was 29.95% (95% CI, 19.1, 40.8%). The presence of heterogeneity was checked by I^2^ statistics. The result of this meta-analysis using the random-effects model revealed that there is high heterogeneity across the included studies (I^2^ = 93%, *P* < 0.01) (Fig. 2).

### Subgroup analysis of non-adherence to inhaled medications by region of the country

**Fig. 3:**
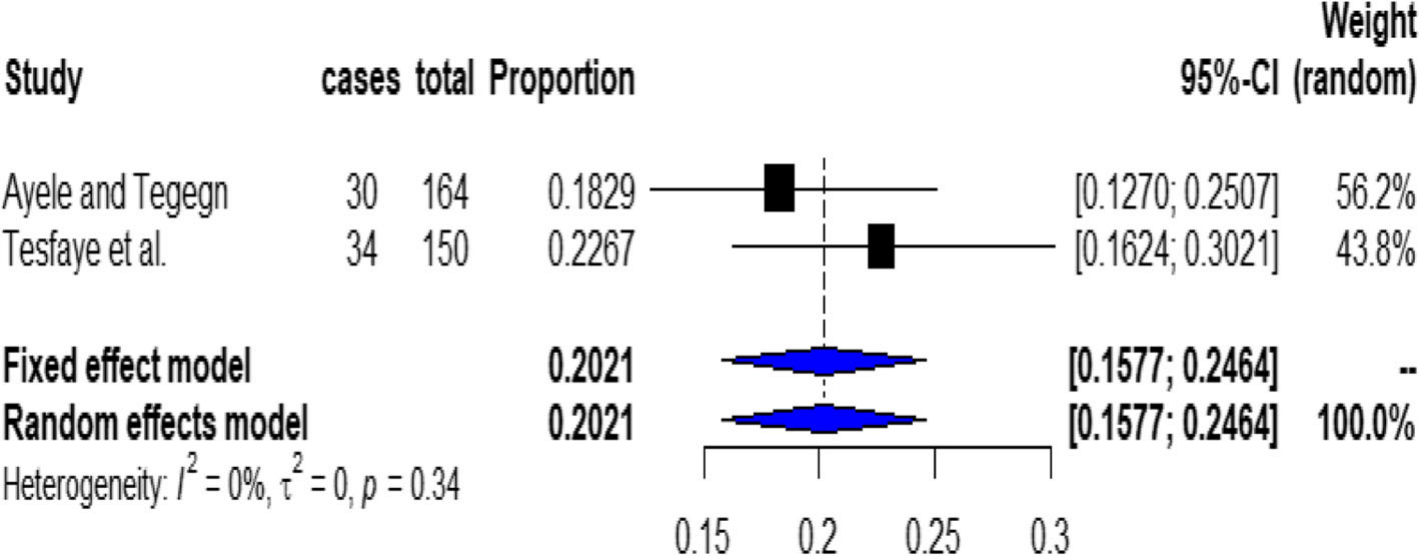
Forest plot for the prevalence of non-adherence to inhaled medications among asthmatic patients in Amhara Region

Based on a subgroup analysis the prevalence of non-adherence to inhaled medications among asthmatic patients in the Amhara region was 20.21% (with 95% CI; 15.77 to 24.64%). The result of this sub group analysis revealed that there is no heterogeneity across the included studies (I^2^ = 0%, *P* = 0.34) (Fig. 3).

**Fig. 4:**
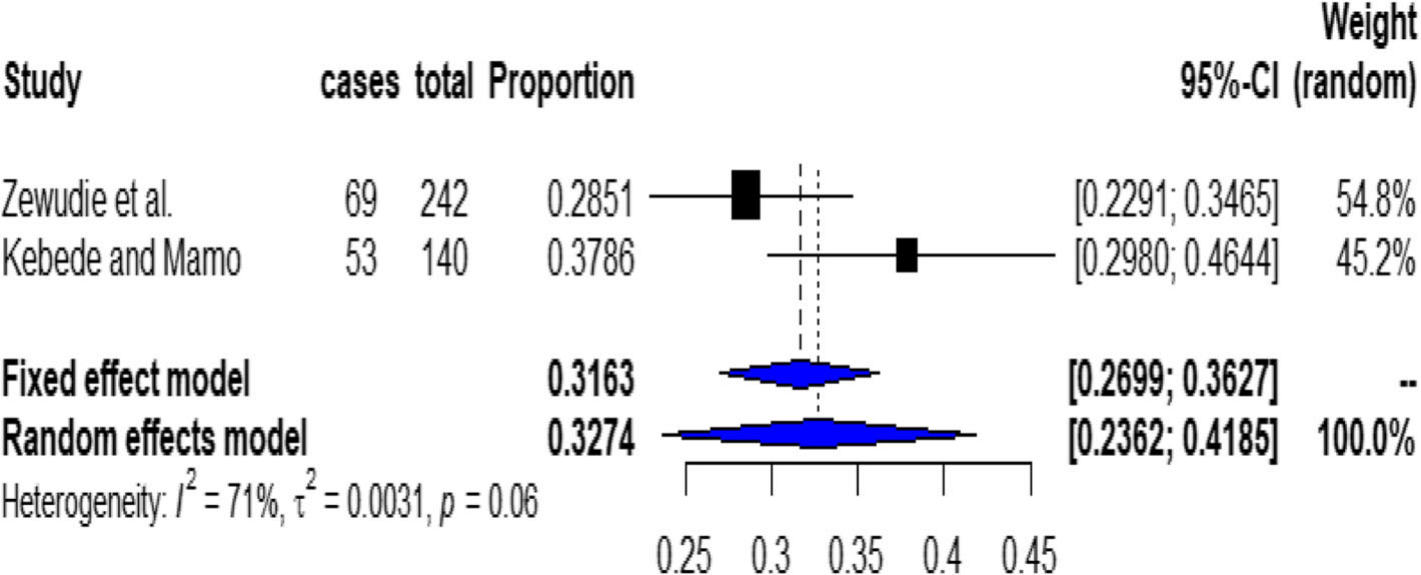
Forest plot for the prevalence of non-adherence to inhaled medications among asthmatic patients in Oromia Region

In this subgroup analysis the prevalence of non-adherence to inhaled medications among asthmatic patients in Oromia region was 32.7% (95% CI; 23.6, 41.85 (Fig. 4).

## Discussion

This meta-analysis was performed to produce pooled estimates of national-wide results of non-adherence to inhaled medications among asthmatic patients. The problem of non-adherence to treatment is the main reason for insufficient asthma control; maximize unwanted drug effects, disease progression, and health care costs. In this study, the pooled national prevalence of non-adherence to inhaled medications was estimated to be 29.95% (95% CI, 19.1, 40.8%). The result of this systemic review and meta-analysis indicated that one out of four asthmatic patients were non-adherent to inhaled medications.

Among the included studies the prevalence of non-adherence to inhaled medications varied from 15.1% reported by Ayele et al. [23] to 59% reported by Koyra and Chinasho [22]. To look at the heterogeneity of the included studies, we conducted subgroup analysis by regions. Based on subgroup analysis, the prevalence of non-adherence to inhaled medications among asthmatic patients in the Oromia (32.7%) was higher than in the Amhara region (20.2%). This indicates that one out of three in Oromia region and one out of five in Amhara region asthmatic patients were non-adherent to their inhaled anti-asthmatic medications.

The finding of this study was lower than the prevalence of single studies conducted in Denmark (61.3%) [33], Northern Ireland (88% in) [34], southeast Michigan (50%) [35], and Kuwait (82.6%) [36]. Whereas this finding is higher than the study conducted in Egypt (21%) [37]. This higher or lower finding might be due to differences in sample size, socio-economic status of the population, fear of side effects, and believes on treatment ineffectiveness for controlling symptoms. Certainly, this evidence was supported by one systematic review study conducted on adherence to treatment with inhaled corticosteroids among asthmatic patients [38]. No meta-analysis study reported the prevalence of non-adherence to inhaled anti-asthmatic medications in international databases. Studies reported that medication-related factors include difficulties with inhaler devices, complex regimens, side effects, cost of medication, dislike of medication, and distant pharmacies were factors are associated with non-adherence to asthma therapy [9, 11, 14, 39].

### Limitations of this study

This is the first systematic review and meta-analysis study drawing the pooled national prevalence of non-adherence to inhaled medications among asthmatic patients. However, the limitation of this study was the failure to identify factors associated with non-adherence to inhaled anti-asthmatic medications. This is due to all most all of the included studies failed to report their results in a cross-tabulation form.

### Conclusion

the prevalence of non-adherence to inhaled anti-asthmatic medications was high. Thus, our finding suggests that one out of four asthmatic patients were non-adherent to inhaled medications.

### Relevance for the clinical practice

This systematic review and meta-analysis informs health care providers’, ministry of health, and health policymakers about the prevalence of non-adherence to inhaled anti-asthmatic medications. This high burden has negative effects to asthma management and outcomes. Therefore, ministry of health, health policymakers, clinicians, and other health care providers should pay attention to strengthening the adherence levels to inhaled anti-asthmatic medications and country-based interventions should be developed to reduce the burden of non-adherence to inhaled anti-asthmatic medications. Reducing this prevalence will have a great contribution on asthma control; increase the quality of life, decrease hospital admission, and mortality of asthmatic patients.

## Supplementary information

**Additional file 1.**

## Data Availability

The data analyzed during the current meta-analysis is available from the corresponding author on a reasonable request.

## Abbreviations

CI: Confidence Interval
PRISMA: Preferred Reporting Items for Systematic Reviews and Meta-Analyses
SNNPE: Southern Nations Nationalities, and Peoples of Ethiopia
Nations: Nationalities, and Peoples, and Ethiopia
